# Genome wide association studies for *japonica* rice resistance to blast in field and controlled conditions

**DOI:** 10.1186/s12284-020-00431-2

**Published:** 2020-10-08

**Authors:** Andrea Volante, Alessandro Tondelli, Francesca Desiderio, Pamela Abbruscato, Barbara Menin, Chiara Biselli, Laura Casella, Namrata Singh, Susan R. McCouch, Didier Tharreau, Elisa Zampieri, Luigi Cattivelli, Giampiero Valè

**Affiliations:** 1 Council for Agricultural Research and Economics-Research Centre for Cereal and Industrial Crops, s.s. 11 to Torino, km 2.5, 13100 Vercelli, Italy; 2 Present Address: CREA Research Centre for Vegetable and Ornamental Crops, Corso Inglesi 508, 18038 Sanremo, IM Italy; 3 Council for Agricultural Research and Economics-Research Centre for Genomics and Bioinformatics, via S. Protaso 302, 29017 Fiorenzuola d’Arda, PC Italy; 4 PTP Science Park, Rice Genomics Unit, via Einstein, 26900 Lodi, Italy; 5 Centre for Sustainable Future Technologies, Istituto Italiano di Tecnologia, Via Livorno 60, 10144 Torino, Italy; 6 SA.PI.SE. Coop. Agricola, via G. Mameli 7, 13100 Vercelli, Italy; 7 School of Integrative Plant Sciences, Plant Breeding and Genetics section, Cornell University, Ithaca, New York, 14850 USA; 8 UMR BGPI, CIRAD, TA A54/K, F 34398 Montpellier, France; 9 BGPI, Université de Montpellier, CIRAD, INRA, Montpellier SupAgro, Montpellier, France; 10 Present Address: Institute for Sustainable Plant Protection, National Research Council, Turin, Italy; 11 Dipartimento di Scienze e Innovazione Tecnologica, Complesso Universitario S. Giuseppe, University of Piemonte Orientale, Piazza S. Eusebio 5, 13100 Vercelli, Italy

**Keywords:** Rice, Blast disease, Genome-wide association study (GWAS), Marker-trait associations (MTAs), Blast resistance

## Abstract

**Background:**

Rice blast, caused by the fungus *Pyricularia oryzae*, represents the most damaging fungal disease of rice worldwide. Utilization of rice resistant cultivars represents a practical way to control the disease. Most of the rice varieties cultivated in Europe and several other temperate regions are severely depleted of blast resistance genes, making the identification of resistant sources in genetic background adapted to temperate environments a priority. Given these assumptions, a Genome Wide Association Study (GWAS) for rice blast resistance was undertaken using a panel of 311 temperate/tropical *japonica* and *indica* accessions adapted to temperate conditions and genotyped with 37,423 SNP markers. The panel was evaluated for blast resistance in field, under the pressure of the natural blast population, and in growth chamber, using a mixture of three different fungal strains.

**Results:**

The parallel screening identified 11 accessions showing high levels of resistance in the two conditions, representing potential donors of resistance sources harbored in rice genotypes adapted to temperate conditions. A general higher resistance level was observed in tropical *japonica* and *indica* with respect to temperate *japonica* varieties. The GWAS identified 14 Marker-Traits Associations (MTAs), 8 of which discovered under field conditions and 6 under growth chamber screening. Three MTAs were identified in both conditions; five MTAs were specifically detected under field conditions while three for the growth chamber inoculation. Comparative analysis of physical/genetic positions of the MTAs showed that most of them were positionally-related with cloned or mapped blast resistance genes or with candidate genes whose functions were compatible for conferring pathogen resistance. However, for three MTAs, indicated as BRF10, BRF11–2 and BRGC11–3, no obvious candidate genes or positional relationships with blast resistance QTLs were identified, raising the possibility that they represent new sources of blast resistance.

**Conclusions:**

We identified 14 MTAs for blast resistance using both field and growth chamber screenings. A total of 11 accessions showing high levels of resistance in both conditions were discovered. Combinations of loci conferring blast resistance were identified in rice accessions adapted to temperate conditions, thus allowing the genetic dissection of affordable resistances present in the panel. The obtained information will provide useful bases for both resistance breeding and further characterization of the highlighted resistance loci.

## Background

Rice blast, caused by the fungal pathogen *Pyricularia* (syn. *Magnaporthe*) *oryzae*, ranked first as the most important rice disease in the world (Dean et al. 2012) determining worldwide annual yield losses of about 4% (Savary et al. 2019). Because of the rapid evolution of new races of the pathogen that causes a breakdown of the deployed resistance (Valent and Khang 2010; Oliveira-Garcia and Valent 2015), blast resistant cultivars (cvs) often have a short field life and typically become ineffective within 2–3 years (Zeigler et al. 1994). Therefore, the introduction of effective and durable resistance to blast in cultivated rice, through marker-assisted selection for stacking different resistance (*R*) genes in the same line, is a priority for rice breeding (Tacconi et al. 2010; Fukuoka et al. 2015; Xiao et al. 2017). To this aim, the identification of new resistance sources is crucial to sustain rice production worldwide.

To date, 500 resistance quantitative trait loci (QTL) and 102 rice *R* genes against *M. oryzae*, called *Pi* (*Pyricularia oryzae*) genes, have been identified in both *indica* and *japonica* rice cvs (Ashkani et al. 2015; Su et al. 2015; Vasudevan et al. 2015; Zheng et al. 2016; cited in Zhu et al. 2016) and among the 102 *Pi* genes, 35 of them have been cloned (Liu et al. 2014; Su et al. 2015; Zheng et al. 2016; Wang et al. 2017). Most of the cloned blast *R* genes encode for nucleotide binding-leucine rich repeats (NB-LRR) proteins (Sharma et al. 2012; Liu et al. 2014; Wang et al. 2014), with exceptions like *Pid2* (Chen et al. 2006) and *Pi21* (Fukuoka et al. 2009) which encode for a lectin protein kinase and a proline-rich protein, respectively.

Linkage Disequilibrium (LD)-mapping based on Genome-Wide Association Studies (GWAS), exploiting marker polymorphisms across all chromosomes, has become increasingly popular and powerful over the last years and widely adopted for QTL mapping in plants (Brachi et al. 2011; Varshney et al. 2014; Ogura and Busch 2015).

GWAS approaches to mine rice blast resistance have been applied in the last years. A GWAS analysis conducted with 151 accessions and a low coverage of SSR markers identified 21 associations with blast resistance, six of which showing co-localization with cloned blast *R* genes (Wang et al. 2015). Moreover, a search for blast *R* genes within a panel of 227 *japonica* genotypes was recently conducted with a low coverage of SSR markers, allowing the identification of 13 significant associations, eight of them ascribable to previously mapped blast *R* genes (Guo et al. 2016). The evaluation of a rice diversity panel (RDP) of 150 tropical *japonica* and 190 *indica* accessions for blast resistance in field conditions identified three significant associations, on chromosomes 1 and 12 for the *japonica* panel and chromosome 8 for the *indica* panel, and candidate genes were discovered on chromosomes 1 and 8 (Raboin et al. 2016). A GWAS for blast resistance in rice was also recently applied using a RDP including 420 accessions representative of the five major *O. sativa* subpopulations that were challenged respectively with five different blast isolates under a growth chamber assay (Kang et al. 2016) and in field conditions in three major rice production areas of China (Zhu et al. 2016). The growth chamber work allowed the identification of 97 loci associated with blast resistance, 82 of which were classified as previously un-identified *R* genes, while the field experiment identified 16 blast resistance loci, 13 of which representing new blast resistance regions. Lin et al. (2018) identified a total of 32 QTLs related to blast resistance, 22 of which co-localized with previously reported resistance *R* genes and/or QTLs, two cloned *R* genes, 19 mapped R loci, and 20 resistance QTLs. Furthermore, a GWAS analysis for blast resistance towards three diverse Chinese isolates conducted using the RDP1 identified 56 QTLs, one of which conferred resistance to all the three isolates and was identified as a new *Pik* allele (*Pik-x*; Li et al. 2019). Finally, the RDP II was recently evaluated for resistance against three *M. oryzae* strains (Liu et al. 2020). The study enabled the identification of 27 loci associated with blast resistance, 22 of which reported as new blast resistance loci, and allowed the identification of a new NB-LRR gene conferring partial resistance to blast. All these works therefore highlighted the effectiveness of the GWAS approach for the identification of new resistance sources for blast in rice.

A survey conducted on 172 temperate *japonica* rice cvs cultivated in Italy and other countries in Europe indicates that only about 9% of them were resistant to three Italian blast isolates (Faivre-Rampant et al. 2011), underlying the need for the identification of blast *R* genes in rice cvs adapted to temperate conditions.

With the aim of identifying new sources of blast resistance within the *japonica* background in terms of both donors of resistance adapted to European growing conditions and new resistance loci, the response to blast of a panel of 279 *japonica* rice accessions was evaluated in field conditions under natural *P. oryzae* inoculum for 2 years. The accessions were high-throughput genotyped by SNP markers and genotypic data associated with the rice blast phenotypic scores and the SNP genotypes were then used for a GWAS for field blast resistance. With the same purpose, a largely overlapping panel (257 accessions in common with the panel evaluated in field conditions) was also evaluated in growth chamber conditions using a mixture of three highly virulent Italian *P. oryzae* isolates (IT02, IT03, IT10; Roumen et al. 1997; Faivre-Rampant et al. 2011; Urso et al. 2016). A total of 14 significant associations were highlighted either in field or growth chamber conditions (3 regions were detected as common in both systems), with three associations putatively representing previously unidentified blast resistance loci. Furthermore, one QTL detected in both the screening procedures, was validated in a biparental mapping population.

## Results

### Evaluation of Blast Response in the Germplasm Collection

A total of 290 and 277 rices, belonging to a panel of 311 *japonica* accessions (including 11 *aus*, *indica* and aromatic cvs as outgroups), were respectively evaluated under field and growth chamber conditions for resistance to rice blast.

**Fig. 1:**
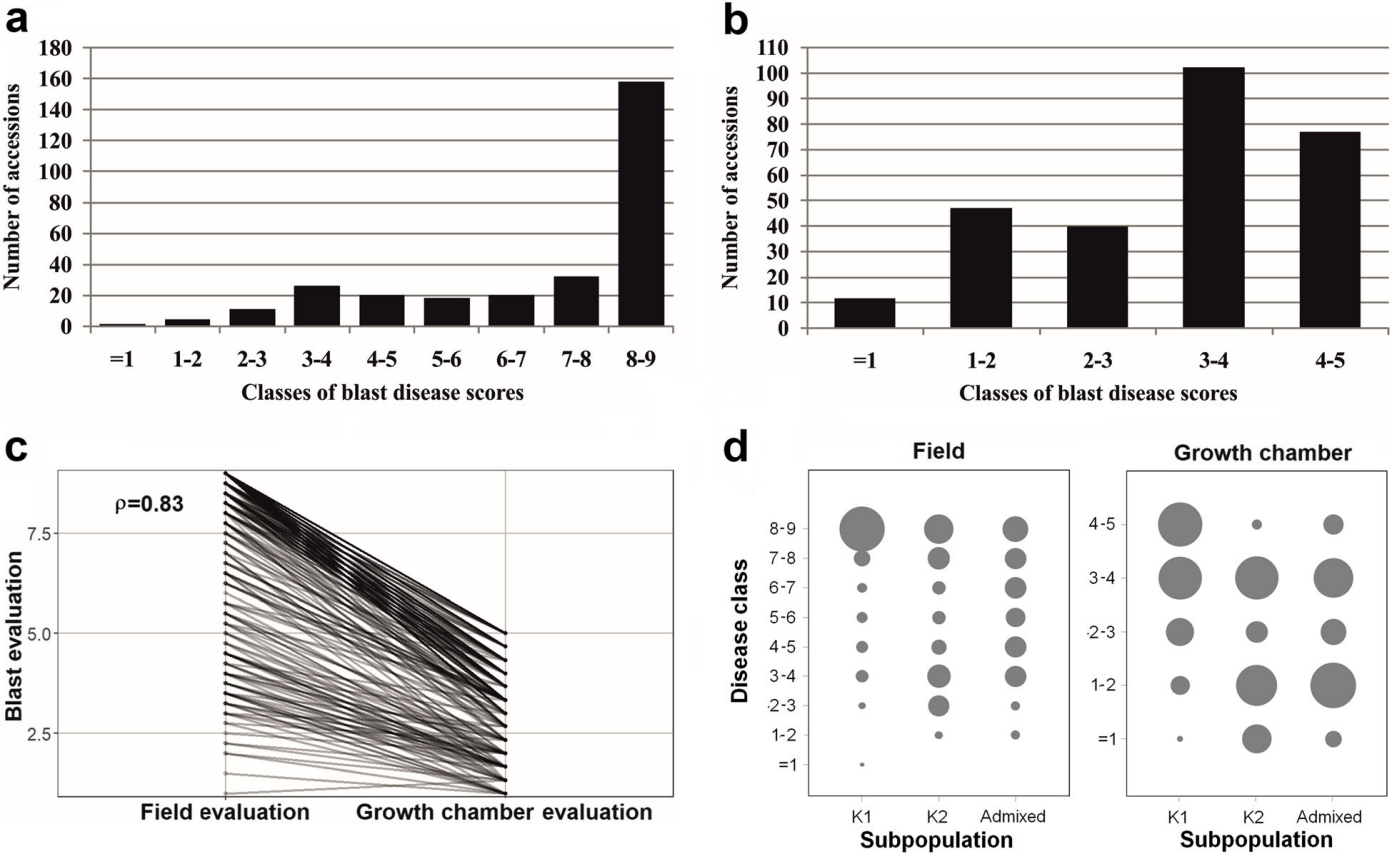
Blast evaluation of the GWAS panel. **a** Frequency distribution of infection types in the field evaluation (1–9 SES scale; International Rice Research Institute (IRRI) 1996. Lower values correspond to higher resistance); **b** Frequency distribution of infection types after growth chamber inoculation with a mix of the three blast isolates (1–5 scale by Faivre-Rampant et al. 2011; as above); **c** Reaction norm between the two infection conditions; Pearson correlation coefficient (ρ) is indicated; **d** Distribution of blast resistance scores of the panel in the three sub-populations

The distributions of blast disease scores for the field evaluations (means of the 2013 and 2014 results) are reported in Fig. 1a. The Pearson correlation value among the average SES scores of the 2 years (means of the two replicates of 2013 and one in 2014) was 0.88 (*p*-value < 2.2e-16), indicating a robust relationship among the data from 2 years. Blast evaluation was based on a 1–9 scoring system on leaves (1 = absence of symptoms; 9 = diseased area > 85%); all the accessions with a score below or equal to 3, showing higher number of small lesions with some becoming elliptical, indicating capabilities in limiting the spread of infection, were considered as resistant, while the remainder as susceptible. Using this threshold, only 5.5% (16/290) of accessions were classified as resistant, while 57.2% (166/290) were highly susceptible (SES scores 8–9; Fig. 1a; Additional file 2: Table S1). The broad sense heritability of blast resistance scores in field conditions was very high (h^2^ = 0.85 calculated on two repetitions; Additional file 3: Table S2) as measured in the 2013 experimental design. Thus, our scoring of blast disease resistance is considered accurate even in absence of replications during 2014.

The 277 rice accessions evaluated under growth chamber conditions were artificially inoculated with a mixture of three Italian blast isolates (IT02, IT03, IT10), showing a broad pattern of virulence towards several blast *R* genes (Roumen et al. 1997; Urso et al. 2016). Based on a 1–5 scoring system on leaves (where accessions with a blast disease score equal to- or below 2 were considered as resistant and the remaining as susceptible), 59 varieties (21.3%) were resistant (Fig. 1b; Additional file 2: Table S1). Seventy-seven accessions out of 277 (27.8%) were highly susceptible (score > 4). The broad sense heritability of blast resistance scores in growth chamber conditions was also high (h^2^ = 0.74; Additional file 3: Table S2).

Pair-wise comparisons of disease resistance between the field and growth chamber data indicate a robust relationship between the recorded phenotypes (Fig. 1c; ρ = 0.83), suggesting a similar response of the panel in the two conditions. Considering the 16 accessions showing field score equal or lower than 3 (Additional file 2: Table S1), 11 (Virgo, Jefferson, GZ6296, Teqing, CNA_4081, Zhenshang_97, Gigante Vercelli, Sakha 103, Giza_178, Lacassine and Venere) also showed growth chamber score lower than 2. For the remaining five varieties, Brazos and Giza 177 yielded a blast score higher than 2 in the growth chamber data, while for other three accessions (IR64, Kristallino and Douradao) growth chamber data were not available. Therefore, of the 59 accessions resistant in growth chamber, only 11 showed resistance in the field, indicating that in field conditions additional blast strains contributed to a higher disease pressure, or that resistance expresses differently in the field. Notably, three accessions (Lamone, Dixiebelle and Giano) with growth chamber score equal or lower than 2, showed field infection scores of 7 (Additional file 2: Table S1).

Based on the evaluation of the disease in the field and growth chamber, the differences in rice blast resistance among genetic sub-populations of the panel were also analyzed (Fig. 1d). The Structure analysis, conducted at increasing K values (as proposed by Courtois et al. 2012) on the whole panel, showed K = 2 as the most probable value (Additional file 1: Fig. S1). At this K value the group of temperate *japonica* accessions (208) was defined, and the percentage of varieties classified as admixed was 12.5% (Additional file 2: Table S1). A principal component analysis (PCA) on genotypic data confirmed this clustering, as the two groups were separated with admixed accessions located in between, while higher K values in the Structure analysis, implemented with a Jukes and Cantor model-based neighbor-joining tree, allowed the expected distinction of tropical *japonica*, and a few *indica*, aromatic and *aus* accessions (Additional file 1: Fig. S1; Additional file 2: Table S1). The median of blast scores in the temperate *japonica* group (K1) were 8.75 and 4.00 for the field and growth chamber evaluations, respectively; while the K2 cluster, containing mainly tropical *japonica*, but also *indica, aus* and aromatic accessions, showed median scores of 5.63 and 2.17 for field and growth chamber evaluations respectively; admixed varieties scored in-between the two main groups (6.00 and 2.17, respectively for field and growth chamber). The differences between temperate *japonica* and the tropical *japonica* plus outgroups were statistically significant, as resulted from Kruskal-Wallis and Dunn’s tests (Additional file 3: Table S2).

#### Analysis of Linkage Disequilibrium and GWAS for Blast Resistance

The SNP panel used in the GWAS consisted of 246,084 markers. After filtering for callrate (> 95%) and minor allele frequency (> 5%) the final set included 37,423 SNPs, with an average value of 3119 markers/chromosome. Assuming an estimated genome size of 373 Mbp (Kawahara et al. 2013), the average density was 9.97 kbp/marker (ranging from 15 for chromosome 3 to 5.9 for chromosome 10). In the GWAS panel, the mean LD decay over the physical distance, computed as r^2^, was 934.2 kbp, ranging from 445 kbp for chromosome 11 to 1355 kbp for chromosome 10 (Additional file 4: Fig. S2).

**Fig. 2:**
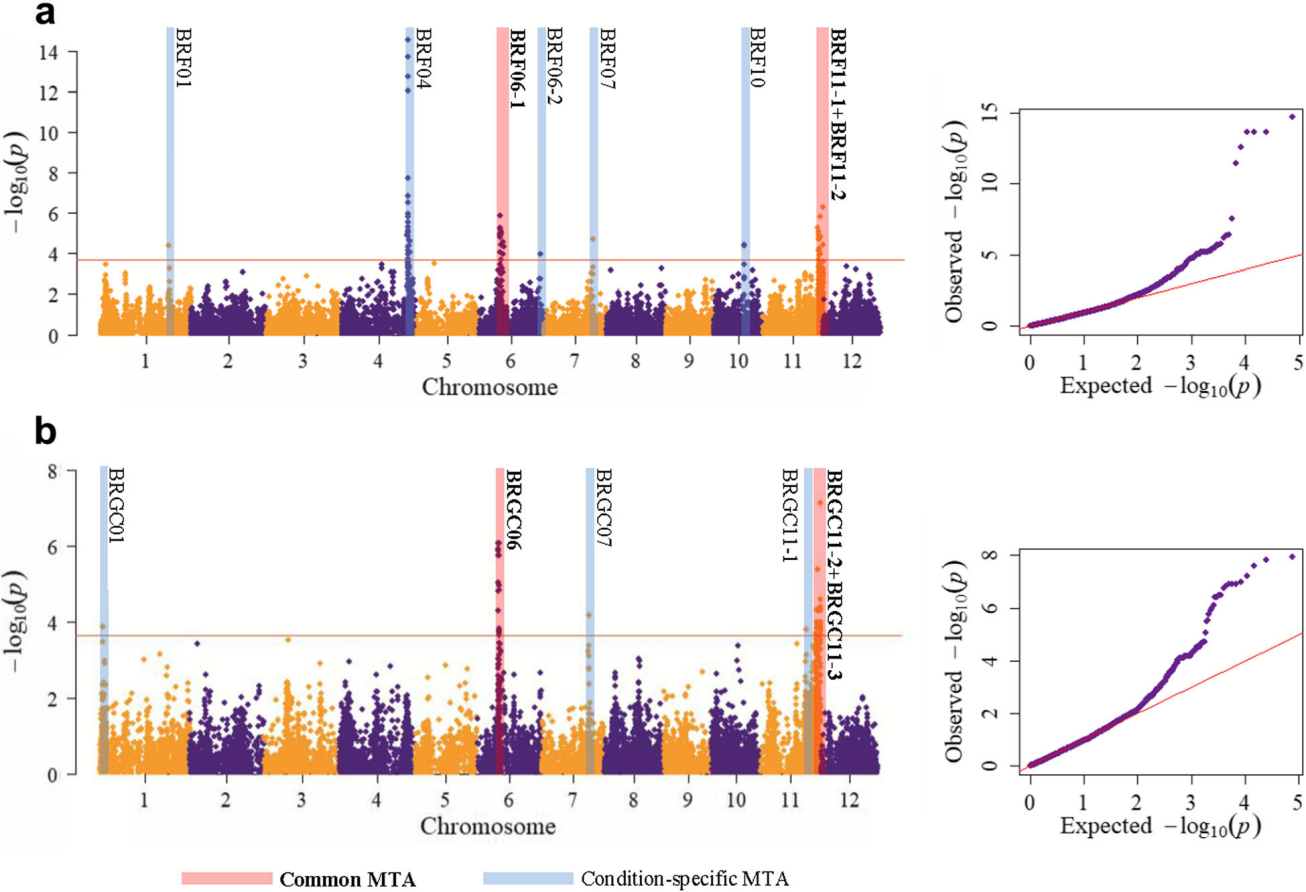
Results of the association analysis for field (**a**) and growth chamber (**b**) evaluations. Left panel: Manhattan plots of the markers associated with rice blast disease resistance. X axis shows markers along the 12 rice chromosomes and Y axis shows the negative log10- transformed *p*-values for each association. MTAs detected in both conditions are red-shaded and named in bold; conditions-specific MTAs are blue shaded. Full redlines indicate the 0.05 FDR threshold. Right panel: Q-Q plots of the –log10(*p*) of the markers from association analyses

The GWAS was conducted considering the disease resistance scores in both field and growth chamber conditions. A total of 14 significant Marker-Trait Associations (MTAs) were identified, with -log_10_(p) of the peak SNP markers ranging between 3.87 and 14.59 and passing a False Discovery Rate threshold of 0.05 (Table 1; Fig. 2). Of these, eight MTAs were discovered for the field data, while six MTAs were detected under growth chamber conditions. In almost all the MTAs, the resistant phenotype was associated with the allele in lower frequency (minor allele). However, the opposite was observed for the BRF06–2 and BRF10 MTAs, for which the accessions carrying the most frequent allele (major allele) displayed lower average blast scores (i. e. higher resistance) with respect to the genotypes with the least frequent allele (Table 1).

**Table 1:** Significant associations between SNP genotypes and blast resistance in the analyzed rice accessions

Category	MTA ID	Peak Marker/Region (bp)	Chr.	-log10(p)	#SNP	Associated region (bp)			Peak marker R^**2** a^	LD block (bp)	SNP alleles at peak marker^b^	Average blast score	Allelic frequence (%)			
						Start	End	Size		Start-End			Total	K1	K2	Adm.
Field	BRF01	S1_32855693	1	4.369	1	–	–	–	6.23%	32,855,693-33,101,889	**A**	7.39	93.5	92.0	100.0	90.9
											G	5.26	6.5	8.0	0.0	9.1
Field	BRF04	S4_31566281	4	14.594	31	31,257,415	32,476,401	1,218,986	19.54%	–	**T**	7.59	91.4	96.4	75.9	91.7
											G	3.23	8.6	3.6	24.1	8.3
Field	BRF06–1	S6_10184497	6	5.889	17	10,108,408	11,927,672	1,819,264	7.77%	–	**C**	7.60	92.4	94.4	92.2	74.2
											A	4.52	7.6	5.6	7.8	25.8
Field	BRF06–2	S6_29136506	6	3.963	1	–	–	–	5.08%	–	**A**	7.06	58.8	57.4	59.6	64.9
											G	7.44	41.2	42.6	40.4	35.1
Field	BRF07	S7_23126442	7	4.723	1	–	–	–	6.18%	–	**A**	7.35	94.5	92.3	100.0	100.0
											G	4.95	5.5	7.7	0.0	0.0
Field	BRF10	S10_14442878	10	4.450	2	14,421,776	14,442,878	21,102	5.79%	–	**G**	6.75	58.6	47.9	79.3	83.8
											A	7.86	41.4	52.1	20.7	16.2
Field	BRF11–1	S11_27464620	11	5.476	28	26,482,681	27,672,972	1,190,291	7.26%	26,090,712-28,791,205	**C**	7.57	91.7	99.5	72.4	83.8
											T	4.12	8.3	0.5	27.6	16.2
Field	BRF11–2	S11_28957771	11	6.304	2	28,900,124	28,957,771	57,647	8.43%	28,807,359-29,018,344	**T**	7.64	83.8	91.3	56.9	86.5
											G	5.05	16.2	8.7	43.1	13.5
Growth chamber	BRGC01	S1_1719121	1	3.872	1	–	–	–	5.65%		**A**	3.48	80.4	97.9	26.4	68.6
											G	2.71	19.6	2.1	73.6	31.4
Growth chamber	BRGC06	S6_10390066	6	6.068	20	9,906,359	10,685,711	779,352	9.55%		**C**	3.51	89.1	94.7	86.5	63.9
											T	2.00	10.9	5.3	13.5	36.1
Growth chamber	BRGC07	S7_22505151	7	4.167	1	–	–	–	6.14%		**G**	3.41	92	90.4	92.5	100.0
											A	2.47	8	9.6	7.5	0.0
Growth chamber	BRGC11–1	S11_25555354	11	4.413	5	24,811,878	25,555,354	743,476	6.03%	24,068,338-26,090,282	**T**	3.54	87	97.3	67.3	61.1
											G	1.95	13	2.7	32.7	38.9
Growth chamber	BRGC11–2	S11_27672972	11	5.975	25	26,482,681	28,167,315	1,684,634	8.34%	26,090,712-28,791,205	**C**	3.47	92.4	97.9	75.5	88.9
											T	1.65	7.58	2.1	24.5	11.1
Growth chamber	BRGC11–3	S11_28957771	11	7.143	34	28,809,430	29,007,377	197,947	11.54%	28,807,359-29,018,344	**T**	3.54	84.1	91.0	58.5	86.1
											G	2.25	15.9	9.0	41.5	13.9

For each trait, the peak marker (SNP with the highest *p*-value) is reported. ^a^variance explained by the marker/region; ^b^Major allele is written in bold letters

Among the MTAs identified in field, the largest allele effects (i.e. the highest difference in average blast scores among the major and minor allele of the peak marker) were observed for BRF04, BRF11–1 and BRF06–1 (4.36, 3.45 and 3.08, respectively), suggesting that gene(s) underlying these MTAs contributed most effectively to blast resistance under these conditions. The same was observed for BRGC11–2, BRGC11–1 and BRGC06 in growth chamber conditions (1.82, 1.59, and 1.51, respectively). For these loci, the highest incidence of favourable alleles at the peak markers was observed in either the K2 (identifying the tropical *japonica* cluster and outgroups) or the admixed group (Table 1). This supports the idea that the blast resistance loci identified, in both field and growth chamber conditions, were mainly derived from tropical and admixed accessions, as already suggested from the analysis of phenotypic scores.

**Fig. 3:**
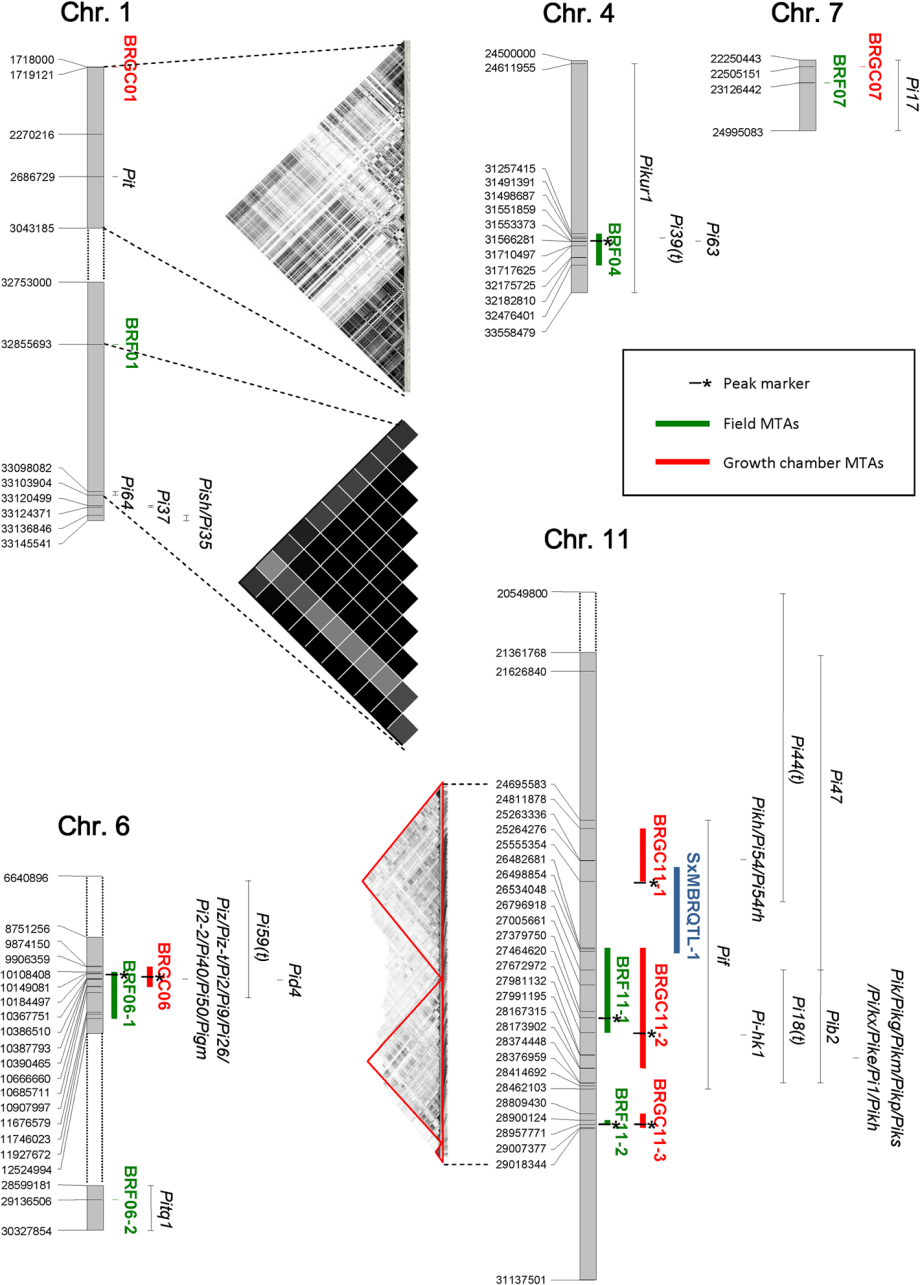
Positional relationships of the significant associations detected in this work, as listed in Table 1 and Fig. 2, with respect to previously mapped or cloned blast resistance (R)-gene regions. BRF = MTAs detected in field conditions; BRGC = MTAs detected in growth chamber conditions. The position of the LD blocks on chromosome 1 and 11 is also indicated

The SNPs showing the most significant *p*-values for the two chromosome 6 MTAs BRF06–1 (from 10,108,408 to 11,927,672 bp) and BRGC06 (from 9,906,359 to 10,685,711 bp) largely overlapped, even considering that the two peak markers were separated by about 205 Kbp from each other (Table 1; Fig. 3; Additional file 5: Fig. S3). On chromosome 11, two regions are defined by largely overlapping MTAs in the two experiments (Table 1; Fig. 3): BRF11–1/BRGC11–2 and BRF11–2/BRGC11–3 (the latter showing a unique peak marker, S11_28957771). Also, in these cases, the data suggest a common origin of resistance for the field and growth chamber experiments.

### Analysis of Candidate Genes at the Blast Resistance Loci

To search for candidate genes underlying the MTAs identified in this study, we considered all annotated genes available from the *Oryza sativa* reference sequence (Os-Nipponbare-Reference-IRGSP-1.0; RAP database: http://rapdb.dna.affrc.go.jp/download/irgsp1.html) and located within the regions defined by −/+ 100 kbp sequences from the MTA peak marker. Moreover, the positions of the significant MTAs were compared with those of known blast *R* genes or QTLs present in literature. Results are summarized in Fig. 3 and Additional file 6: Table S3.

The genomic intervals identified by BRF06–1/BRGC06, BRF11–1/BRGC11–2 and BRF11–2/BRGC11–3 overlapped in both field and growth chamber assays. For BRF06–1/BRGC06, the interval between the two peak markers (from 10,184,497 to 10,390,066 bp) was treated as a single peak. The search on RAP-DB detected a total of 26 genes located in the chromosome 6 interval from 10,084,497 to 10,490,006 bp. Of these, 20 were functionally annotated and included Os06g0286700 (from 10,387,793 to 10,390,465 bp), corresponding to the cloned blast *R* gene *Piz* and its allelic variants *Piz-t, Pi2, Pi9* and *Pi40* (Zhou et al. 2006; Wang et al. 2017)*,* and Os06g0287500, corresponding to *Pid4* (from 10,435,819 to 10,441,907 bp; Chen et al. 2018) both closely proximal to the BRGC06 peak marker (at 10,390,066 bp; Fig. 3). Two additional cloned blast *R* genes, *Pi59(t)* and *Pigm(t),* were also present in this region (Fig. 3).

Thirty-six genes were detected in the chromosome 11 region analyzed for BRF11–1/BRGC11–2 (27,364,620-27,772,972 bp; Additional file 6: Table S3). Three of them (Os11g0678400, Os11g0684100, Os11g0684700) corresponded to NB-ARC containing proteins. These types of sequences have been described as part of the catalytic domain of R proteins and show a high grade of polymorphism in different alleles of the *Pib* gene (van Ooijen et al. 2008; Vasudevan et al. 2015). Literature searching highlighted that a large cluster of *Pi* genes is localized in a region overlapping BRF11–1/BRGC11–2 (Fig. 3). This group includes, among the others, *Pik* (Os11g0689100; 27,984,697-27,989,128 bp), as well as its allelic forms (*Pik-m, Pik-p, Pik-s, Pik-g* and the two recently described alleles *Pik-e* and *Pik-x*; Chen et al. 2015; Li et al. 2019). However, none of the considered *R* genes was coincident with the peak markers of the MTAs.

No genes with a function clearly related to blast resistance were detected in the region analyzed for the MTAs BRF11–2/BRGC11–3 (28,857,771-29,057,771 bp; Additional file 6: Table S3).

The remaining MTAs were specific to either growth chamber or open field conditions. In the BRF01 region (32,755,693-32,955,693 bp), no genes having a function related to pathogen resistance were detected using RAP-DB, with the exception of a MATH domain containing protein (Os01g0775300; Additional file 6: Table S3). This type of protein is a pathogen-responsive membrane bound receptor kinase, involved in the early phases of fungal interaction in *Arabidopsis* (Peškan-Berghöfer et al. 2004; Shahollari et al. 2005). However, BRF01 is a single-marker MTA located approximately 242–290 Kbp from a cluster of *Pi* genes: *Pi64* (Os01g0781200), *Pish* (Os01g0782100) and *Pi37* (Os01g0781700). Local LD decays slowly in this region, as evidenced by a 246 Kbp block, spanning from 32,855,693 to 33,101,889 bp, and covering the entire region between BRF01 (32,855,693 bp) and *Pi64* (33,098,082-33,103,904 bp) (Fig. 3). The remaining two genes lie in a region spanning approximately 101 Kbp, covered only by monomorphic markers in our dataset, therefore no further information can be provided; nevertheless, we cannot exclude the possibility that the LD block spans the whole *Pi* gene cluster in this region.

Twenty-six genes, 18 of which were functionally annotated, were detected in the BRF04 locus (31,157,415-32,576,401 bp; Additional file 6: Table S3). This list included an ABC-transporter (Os04g0620000) and a disease resistance domain containing protein (OsO4g0621500). The latter was located at about 10 Kbp from the peak marker, raising a possible involvement in blast resistance governed by BRF04 for this gene. Moreover, co-positional relationships were observed for BRF04 and the *Pi39(t)* and *Pi63* blast resistance loci (Fig. 3).

The BRF06–2 region contained 27 genes, of which 21 with known functions. Among them, Os04g0695600 encodes a RING protein with ligase E3 function. This class of enzymes is potentially involved in *Magnaporthe grisea* resistance in rice by influencing cell wall properties and the accumulation of hydrogen peroxide and phenolic compounds (Li et al. 2011). Moreover, the BRF06–2 region was positionally related to *Pitq1* (28,599,181-30,327,854; Sharma et al. 2012; Fig. 3).

Thirty-five genes were detected in the region investigated for BRF07 candidates, including Os07g0573100 and Os07g0573200, both encoding adenosine-sulphate kinases which play a role in the synthesis of sulfur compounds, such as glutathione, which are involved in the stress response and resistance mechanisms (Wang et al. 2016). Os07g0563300is also localized in the BRGC07 region (22,405,151-22,605,151 bp) and corresponds to a B3 domain transcriptional repressor which regulates the mechanisms of resistance to *phytofagous* insects in rice by activating the salicylic acid signalling pathway and suppressing the jasmonic acid/ethylene-dependent pathway (Wang et al. 2015). These mechanisms could have a low specificity of action, and thus be implicated in resistance to fungal pathogens. Both BRF07 and BRGC07 partially overlap with the region where the blast *R* gene *Pi17* was mapped (22,250,443-24,995,083 bp; Sharma et al. 2012; Fig. 3).

The chromosome 1 BRGC01 included Os01g0132100, encoding for a leucine-rich repeat containing protein. No cloned or mapped blast *R* genes were present within this region; however, an LD block spanning the whole interval (approximately 500 Kbp) contained both BRGC01 and the *Pit* gene (Fig. 3).

The region surrounding BRGC11–1 contained 24 genes. Fifteen of them have a known function. Four genes have an annotation correlated with disease resistance: Os11g0644100 (Leucine-rich repeat N-terminal domain containing protein), Os11g0647600 (Plant disease resistance response-related protein) and Os11g0648600 and Os11g0649100 (both NB-ARC domains containing genes). Moreover, the locus is positionally related to *Pikh* (and its allelic form *Pi54*; Sharma et al. 2005).

Finally, none of the three BRF10, BRF11–2 and BRGC11–3 MTAs colocalized with R genes previously identified using a traditional mapping strategy; we also compared the three loci with map position of previously reported major rice blast resistance loci (Ashkani et al. 2015; Su et al. 2015; Vasudevan et al. 2015; Zheng et al. 2016) including 35 cloned genes (Liu et al. 2014; Su et al. 2015; Zheng et al. 2016; Wang et al. 2017), and no overlap regions were found. However, within the 200 Kbp interval surrounding the BRF10 peak marker (14,442,878 bp), 21 genes were annotated, 17 of which have a known function (Additional file 6: Table S3). Among them, Os10g0413400 encodes a Glycerol–3–phosphate acyltransferase, an enzyme homologous to *AtGPAT6*, involved in *Arabidopsis* resistance to fungal pathogens (Chanda et al. 2008). The BRF-10 resistant allele is present in the durable blast resistant accession Gigante Vercelli (Urso et al. 2016), together with BRF01 and BRF04, suggesting that it could represent a useful resistance locus. The region associated to BRF11–2/BRGC11–3 (28,857,771-29,057,771 bp) included 22 annotated genes, none of which showing functions that could be clearly related to blast resistance (Additional file 6: Table S3). As discussed below, BRF11–2 and BRGC-11-3 co-localize; they share the same peak marker and within resistant accessions respectively represent the second most frequent allele in field conditions and the most frequent allele in the growth chamber (Additional file 2: Table S1), suggesting an important and useful role in blast resistance. Considering that for these three MTAs no obvious candidates or positional relationships with previously reported blast resistance genes could be identified, the obtained results raise the possibility that these MTAs represent novel blast resistance loci.

### Validation of BRGC11–1 on Chromosome 11 in a Biparental Segregating Population

BRGC11–1 was an MTA providing a high allelic effect (i.e. blast score difference between major and minor allele: 1.59) under growth chamber conditions. To further dissect and validate the role of this region in blast resistance, a biparental F_2_/F_3_ segregating population derived from across between the resistant accession Salvo (showing the resistant allele G at BRGC11–1) and the susceptible accession Maratelli (showing the susceptible allele T; Additional file 2: Table S1), was used. The disease reactions of Salvo and Maratelli and the 130 F_2_/F_3_ lines were subjected to artificial inoculation with a mixture of three blast isolates (IT02, IT03, IT10) under growth chamber conditions. Infection and disease scores were evaluated following the procedures adopted for the GWAS of the 277 accessions. The analysis of variance of phenotypic data revealed a highly significant effect of the genotype (*P* < 0.0001) on the disease incidence, with no significant differences observed between replications (*P* = 0.38). No transgressive lines (with extreme resistance/susceptibility phenotypes) were observed, suggesting that resistance factors were contributed only by Salvo. The estimated broad sense heritability for resistance within the Salvo x Maratelli population was 92.5%, indicating that most of the phenotypic variance was due to genetic effects.

A total of 3724 SNP markers together with 46 SSRs were used to assemble the genetic linkage map. After elimination of the unlinked loci, the genotypic data relating to 1581 informative marker loci were assembled into 15 linkage groups corresponding to the 12 rice chromosomes (Additional file 8: Table S5A). Two linkage groups were identified for chromosomes 2, 4 and 5. The overall length of the map was 1755.2 cM with individual chromosome genetic length ranging from 120.1 cM (chromosome 6) to 187 cM (chromosome 5) and average chromosome length of 146.27 cM. The total number of mapped loci per chromosome ranged from 67 (chromosome 8) to 226 (chromosome 1) with an average of 131.75 loci per chromosome. The genome-wide marker density was 1.11 cM/marker, varying from 0.68 cM/marker (chromosome 1) to 2.25 cM/marker (chromosome 8) (Additional file 8: Table S5B).

The searching for blast resistance associated loci allowed the identification of one highly significant QTL (SxMBRQTL-1) on chromosome 11, with a LOD score of 29.81 and explaining 74% of the phenotypic variation. The QTL peak was represented by the RM224 SSR marker and, based on Darvasi and Soller (1997), the confidence interval covered a region of 5.85 cM, from SNP S11_25478006 to SNP S11_26583984. The negative value of the additive effect suggests that the resistance allele at this locus was derived from Salvo (Additional file 8: Table S5C). The region defined by this QTL overlapped with the BRGC11–1 peak marker (S11_25,555,354, 25,555,354 bp), and partially overlapped with regions defined by BRF11–1 (26,482,681-27,672,972 bp) and BRGC11–2 (26,482,681-28,167,315 bp).

## Discussion

### Screening of the GWAS Panel Highlighted Resistant Accessions and Differential Resistance Levels

Most of the *japonica* rice cvs cultivated in temperate regions are susceptible to blast, a situation that underlines the relevance of identifying suitable sources of resistance adapted to these environments and loci governing the traits to be used in marker-assisted breeding for durable blast resistance. This study identified sources of resistance adapted to temperate conditions and with limited barriers for crossing with temperate *japonica*. The adoption of the two screening procedures allowed to discriminate accessions *(i. e.* Lamone, Dixiebelle and Giano) showing resistance under the simplified pressure of a mixture of three blast isolates but being susceptible under a different or more complex pathogen field population. Moreover, the application of two different disease pressures allowed the identification of a high number of genomic regions involved in blast resistance, thus boosting the power of the investigation. Indeed, of the 14 MTAs identified, five (BRF01, BRF04, BRF06–2, BRF07 and BRF10) were detected only under field conditions while three (BRGC01, BRGC07 and BRGC11–1) only after inoculation in growth chamber conditions. Similarly, previous GWAS studies for blast resistance conducted using the rice diversity panel 1 (RDP1; Eizenga et al. 2014) screened in the growth chamber with five diverse *M. oryzae* isolates (Kang et al. 2016) and in field conditions (Zhu et al. 2016) led to the identification of non-overlapping resistance loci, supporting the hypothesis that different resistance mechanisms can be highlighted using these different screening procedures (Zhu et al. 2016).

The Structure analysis identified two subpopulations at K = 2 corresponding to temperate (208 accessions) and 53 tropical *japonica* genotypes with 11 outgroups (64 accessions), while admixed represented the 12.5%. When the disease scores were analyzed in relation to each group, the average disease scores were higher for the temperate *japonica* accessions compared to the remaining accessions (Fig. 1d). The result confirms that a range of diversity, in terms of disease resistance, was captured in the present panel, and that a high level of quantitative resistance based on preformed (i.e. before infection) defense expression exists in the tropical *japonica* group, as previously stated (Vergne et al. 2010; Raboin et al. 2016). In agreement with our observations, also a recent screening of the RDP1 panel performed using both inoculation with blast isolates and under field conditions, highlighted a higher level of blast resistance in tropical *japonica* with respect to admixed, *aus*, *indica,* aromatic and temperate *japonica* sub-populations (Kang et al. 2016; Zhu et al. 2016). The few *indica* accessions included in the panel, although much less numerous than the other groups, showed a generally higher level of resistance: the median disease scores were 1.33 in the growth chamber (compared to 2.00 and 4.00 for tropical and temperate *japonica*, respectively) and 2.25 in the field (6.50 and 8.75 for tropical and temperate *japonica*, respectively). This observation is in agreement with evidence that several donors for qualitative *Pi R* genes conferring high levels of vertical resistance belong to the *indica* group (Sharma et al. 2012; Liao et al. 2016; Raboin et al. 2016).

### The GWAS Allowed the Identification of Effective Blast Resistance Loci in Rice Accessions Adapted to Temperate Conditions

The mean genome-wise LD (r^2^) observed In the GWAS panel decayed to 0.2 (one of the most commonly used thresholds to define unlinked loci; Vos et al. 2017) at 934.2 kbp. Although such a decay value is higher than those generally present in literature (Mather et al. 2007), similar or even higher LD values in rice populations were previously highlighted (Kumar et al. 2015; Xu et al. 2011). Overall, the discrepancies between decay values reported in literature are mainly related to differences in the genetic materials (genetic diversity) and in the number of markers used for the analyses (Volante et al. 2017a). Nevertheless, our results suggested that the panel has a sufficient detection power for an association mapping approach.

The GWAS experiment yielded a total number of 14 MTAs putatively involved in resistance to blast disease. Some of the MTAs explained larger proportions of phenotypic variation than others, such as BRF04, BRF11–1 and BRF06–1, associated with field blast resistance, and BRGC11–2, BRGC11–1 and BRGC06, associated with resistance in growth chamber. Such behavior would suggest that these loci are the most effective in controlling multiple pathogen strains (BRF) or a mixture of a restricted number of strains (BRGC). Furthermore, since the positions of BRF11–1/BRGC11–2 and BRF06–1/BRGC06 are overlapping, these loci should be considered effective in governing both single and multiple strain resistance and can be suggested as the most interesting and useful targets for blast resistance breeding. For example, resistant alleles at both loci are present in accessions like Arsenal, Cadet, Jefferson, and UPLA_91, and these lines could therefore be used as resistance donors in breeding programs, using the polymorphisms at the peak markers of the identified blast resistance associated regions for molecular marker assisted selection.

Analysis of haplotypes around peak markers in the 11 accessions showing resistance in both phenotyping conditions (Virgo, Jefferson, GZ6296, Teqing, CNA_4081, Zhenshang_97, Gigante Vercelli, Sakha 103, Giza_178, Lacassine and Venere; Additional file 2 Table S1) differed between resistant and susceptible cvs and can provide useful information about their exploitation in blast resistance breeding. Interestingly, the resistance in *indica* accessions (e.g. GZ6296, CNA4081 and Giza_178) under both field and growth chamber conditions was associated with a conserved combination of MTA resistant alleles (that included BFR04, BRF06–2, BRF11–2, BRGC01 and BRGC11–3), whereas for temperate and tropical *japonica,* high levels of resistance were achieved through substantially different combinations of MTA alleles. For example, Virgo (temperate *japonica*, the most resistant cv in field conditions) carries resistant alleles at BRF01, BRF06–1, BRF07 and BRGC07; Jefferson (tropical *japonica*) has resistant alleles at most of the MTAs; Gigante Vercelli carries resistant alleles at BRF04 and BRF10. The BRF04 resistant allele is the most frequently identified (and the most effective) in field conditions and its position (31,257,415 to 32,476,401 bp) is adjacent to a QTL for durable blast resistance qBDR4 (32,499,412 to 32,887,572 bp) identified in a cross between the durable resistant Gigante Vercelli and the susceptible Vialone Nano (Urso et al. 2016). BRF04, putatively conferring durable resistance, appears to have been selected in several temperate and tropical *japonica*, as well as in *indica* accessions, adapted to temperate conditions. For the temperate *japonica* Sakha 103, resistant alleles are present for BRF06–1 (and BRGC06), BRF07, BRF10, BRGC07 and BRGC11–3, while for the tropical *japonica* Lacassine, resistant allele were highlighted for BRF06–2, BRF11–1, BRF11–2 and BRGC11–1/2/3. Finally, the temperate *japonica* Venere brings resistant alleles for BRF07 and BRGC07. The use of these accessions as blast resistance donors in breeding can be assisted with the identified polymorphisms at the MTAs by selecting the resistant alleles for all the involved loci or just selecting those providing larger values of explained phenotypic variance and larger gap in average blast scores among the major and minor allele of the peak marker.

As described in the Results section, a comparative analysis of the physical/genetic positions of the MTAs detected in this study with cloned or mapped blast *R* genes identified in previous studies highlighted many co-positional relationships. In particular, clusters of known *Pi* genes were located within the MTA regions identified using both the screening procedures on chromosomes 6 and 11. In other cases, although the positions did not exactly overlap, local LD indicated that both the MTAs and *Pi* genes were co-located in unique linkage blocks, raising the possibility that the resistance effects measured in this study were associated with previously cloned/mapped *Pi* genes.

BRGC06 colocalizes within the resistance gene *Piz* and its allelic forms, while BRF06–1 is in close proximity (Fig. 3; Additional file 7: Table S4); the frequency of the resistant alleles at these loci were higher in the varieties classified as resistant (33% and 35% in field and growth chamber, respectively) than in the susceptible ones (7% and 6%). Jefferson, Zenith and Bengal are known in literature to harbour the *Piz* *R* gene (Tacconi et al. 2010) and, except for Bengal, showed blast resistance in this work. Therefore, *Piz* can be suggested as the most probable candidate gene for this region. The *Pid4* blast resistance gene (Os06g0287500) is also localized on chromosome 6, inside the BRF06–1 and BRG06 MTAs (from 10.435.819 to 10.441.907 bp; Chen et al. 2018). *Pid4* encodes a a typical CC-NB-LRR protein localized in both nucleus and cytoplasm and was constitutively expressed in uninfected plants (Chen et al. 2018).

Additional fine mapping experiments using larger biparental populations and/or allele mining based on the sequencing of accessions in a diversity panel would be required to further dissect the relationships among MTAs and *Pi* candidates. However, for three MTAs (BRF-10, BRF11–2, BRGC11–3) there are no obvious resistance gene candidates, likely indicating that these loci might harbor previously unidentified blast *R* genes. A survey of the Nipponbare annotated genome was performed, by screening a region (peak marker of each MTA −/+ 100 Kbp) approximately corresponding to a LD decay to its half-maximum as a trade-off between accuracy and power of the analysis; this allowed to restrict the analyzed interval and to focus on stronger marker-gene associations. Several genes were thus identified in the peak regions of these MTAs, whose function is related to mechanisms of resistance to biotic stress, suggesting that they may contribute to the resistant phenotypes. These genes include a MATH domain containing protein, a RING protein with ligase E3 function, adenosine-sulphate kinases, a B3 domain transcriptional repressor, a glycerol–3–phosphate acyltransferase, a Leucine-rich repeat N-terminal domain containing protein, a plant disease resistance response-related protein, and two proteins containing NB-ARC domains. Interestingly, a pair of co-located loci, the BRF11–2 and BRGC-11-3, were identified using the two different phenotypic screening procedures, and the BRF11–2 resistant allele is the second (after BRF04) most frequent allele detected in resistant accessions under field conditions, while the BRGC11–3 resistant allele is the most frequent allele detected in resistant accessions in the growth chamber (Additional file 2: Table S1). These results suggest that this locus has been under selection in temperate and tropical *japonica* varieties, as well as in temperate-adapted *indica* accessions, where blast disease is a serious problem.

Overlapping was observed with recently described but not yet fully characterized genomic regions associated with blast resistance. Ding et al. (2020) conducted a genome-wide sequence analysis associated to an expression study of rice NLR genes responsive to *Xanthomonas oryzae* and *Magnaporthe oryzae* and identified a total of 46 pathogen-responsive NLR genes. Two of them, LOC_Os11g42660 (Os11g0646300, position bp 25,695,249-25,699,181) and LOC_Os11g45930 (Os11g0686500, position bp 27,793,778-27,797,821), were located inside the LD blocks of BRGC11–1 (bp 25,555,354) and BRF11–1/BRGC11–2 (bp 27,464,620-27,672,972), respectively. Overlapping was also observed between the physical positions of the resistance QTLs identified in the present work and the ones discovered by Li et al. (2019). More specifically, co-positional relationships are present for QTL21 (bp 10,453,060) and BRF06–1/BRG06 (bp 10,184,497/10,390,066), QTL48 (bp 27,610,985) and BRF11–1 (bp 27,464,620). Moreover, co-positional relationships were also discovered for BRF06–1 (bp 10,184,497) and BRGC06 (bp 10,390,066) with MTAs named as seq-rs2910 (bp 10,953,758) and seq-rs2916 (bp 11,106,587), identified as associated with resistance to 16 blast strains within a set of 355 *indica* rice accessions (Lu et al. 2019).

Recently, Liu et al. (2020) detected 27 loci associated with rice blast resistance (LABRs) following a GWAS approach on the Rice Diversity Panel II (C-RDP-II); four of them overlapped with our MTAs. As first, LABR15 corresponded to a SNP on chromosome 6, located at 10,282,193 bp, inside of BRF06–1 and BRGC06. Most interesting, three LABRs were located on chromosome 4 (LABR11–bp 31,159,185, LABR12 – bp 31,381,117 and LABR13 – bp 32,143,028), at the level of BRF04 (bp 31,566,281), and, for LABR12 an NLR gene cluster was discovered (Liu et al. 2020). One of the NLR genes belonging to this cluster, LOC_Os04g53050, was conserved in different partially resistant rice cvs and its expression was activated at early stages of blast infection. This gene, defined as *Partial Resistance gene 1* (*PiPR1*), represents a non-strain specific partial *R* gene (Liu et al. 2020) and can be also considered as a candidate for blast resistance associated to the BRF04.

Finally, in the present work a linkage mapping approach allowed to detect a single QTL on chromosome 11 (SxMBRQTL-1) in a region that is replete with known blast resistance genes. SxMBRQTL-1 was sparsely covered by SNPs in the diversity panel, yet it showed a correspondence with the BRGC11–1 interval and, most importantly, the peak marker of BRGC11–1 was located within SxMBRQTL-1. Approximately 50% of the resistant cvs analyzed in growth chamber experiments were found to carry the resistant allele at this locus. These results suggest that chromosome 11 harbors a large reservoir of genetic variation that contributes to blast resistance in temperate-adapted rice varieties.

## Conclusions

The parallel screening (field and growth chamber) for blast resistance of this study allowed the identification of blast resistant rice accessions adapted to temperate conditions. The adoption of the two screening procedures uncovered accessions showing differential resistance under the two conditions and allowed us to identify a larger number of genomic regions conferring blast resistance than relying on a single approach. The GWAS approach identified 14 MTAs associated to blast resistance. Five of them were detected only under field conditions, while three were discovered only for the growth chamber experiment. The screening procedures identified the largest number of resistant accessions in the cluster of tropical *japonica* and outgroup varieties, though highly resistant temperate *japonica* genotypes were also identified.

Some of the 14 MTAs explained a larger proportion of phenotypic variation than others, and MTAs providing resistance to multiple pathogen strains in the field often overlapped with MTAs providing resistance to a restricted number of strains under growth chamber conditions. Loci conferring high level of resistance in both conditions provide useful targets for blast resistance breeding. When resistant alleles at the MTAs were analyzed with respect to sub-populations, it was observed that the *indica* accessions with high levels of resistance shared a conserved combination of MTA resistant alleles, while the temperate and tropical *japonica* cvs achieved high levels of resistance based on a variety of different combinations of resistant alleles at MTA peak markers.

Previously cloned or characterized blast *R* genes and recently described QTLs were found to be co-located with a large proportion of MTAs, providing numerous “candidates” underlying the associations reported in this study. However, for BRF-10, BRF11–2, BRGC11–3 no obvious relationships with cloned/mapped *Pi* genes was found, raising the possibility that these loci may represent previously unidentified genetic sources conferring quantitative resistance to the rice blast disease.

## Methods

### Plant Materials

The panel used in this study, largely overlapping with the one described in Volante et al. (2017a), is composed of 311 *Oryza sativa* accessions from the Rice Germplasm Collection maintained at CREA-Research Centre for Cereal and Industrial Crops (Vercelli, Italy). This panel is composed of 76 tropical *japonica*, 224 temperate *japonica*, 8 *indica*, 2 aromatic and 1 *aus* accessions. Most of these accessions (162) were selected in Italy, 35 in United States, 27 in Portugal, 17 in Spain, 10 in Bulgaria, 10 in Argentina, 8 in France, and the remaining were developed elsewhere but considered as well adapted to Italian agro-climatic conditions. Detailed information regarding the accessions is reported in Additional file 2: Table S1.

A segregant population of 130 F_2_/F_3_lines was obtained from a cross between the blast resistant accession Salvo and the blast susceptible accession Maratelli, both present in the rice panel described above. Young leaf tissue from each F_2_ plants and the two parents were utilized for the extraction of genomic DNAs through the DNeasy Plant Mini Kit (QIAGEN) following manufacturer’s instructions.

### Inoculation and Evaluation of Blast Resistance

For field infection, the panel was evaluated for blast disease resistance in two field seasons in 2013 and 2014. The experiments were conducted in a breeding station in North west of Italy (45° 9′ 17,28″ N, 8° 49′ 51,24″ E) at an altitude of 96 m asl. Blast infection occurred naturally, and spreader rows of the highly susceptible variety Maratelli, sown on the plot borders, were used to ensure high, homogeneous disease pressure. Moreover, to increase blast incidence, a delayed sowing (middle of July) was performed in both experiments. A total of 290 accessions were evaluated in each field season; for each accession, two lines of 2.4 m were sown for a total of 120 plants per accession. Two replicates were performed in the 2013 trials, while no replications were conducted in 2014. Disease scoring was evaluated at full tillering stage using a 1–9 scoring system (1 = no symptoms; 2 = a few very small lesions; 3 = higher number of small lesions some of which become elliptical; 4 = expanding lesions with a total lesion area of up to 5%; 5 = lesion area 5–10%; 6 = lesion area 15–30%; 7 = lesion area 30–60%; 8 = lesion area 60–85%; 9 = lesion area > 85%) modified from the Standard Evaluation System (SES) for Rice (International Rice Research Institute (IRRI) 1996).

A pathogenicity seedling test under controlled conditions was conducted to evaluate the blast response of 278 accessions belonging to the rice panel and 130 F_3_ lines derived from the cross between Salvo x Maratelli. Plants were grown from seeds in growth chamber and the reaction to blast was tested with three different Italian *M. oryzae* isolates (IT02, IT03, IT10; Roumen et al. 1997), obtained from the UMR BGPI CIRAD collection (Montpellier, France). The selected strains, collected from different geographic regions and isolated from single spores, represent three different lineages with a peculiar virulence pattern, as previously described (Faivre-Rampant et al. 2011; Urso et al. 2016). A complete randomized design was applied to the inoculation experiment. Rice plants were grown as previously described (Urso et al. 2016), subsequently, 20 days old plantlets of the 278 accessions and the F_3_ lines were inoculated with a mix of the three isolates. Fungal cultures were prepared for spray inoculation as previously described (Faivre-Rampant et al. 2011; Urso et al. 2016). Seven days after inoculation, macroscopic phenotypes (necrosis and lesions) on leaves were recorded according to the infection scale derived from Roumen et al. (1997) and described in Faivre-Rampant et al. (2011), with scores from 1 to 5 (1–2 = resistant type lesions without sporulation; 3 = small sporulating type lesions with a grey centre and a brown margin; 4–5 = susceptible extended sporulating lesion type with or without a brown margin). Leaf ratings 1 and 2 were associated to resistance, while ratings 3–5 were associated to susceptibility. For the accessions belonging to the panel, three independent fungal inoculations of ten plants each were performed and the mean value was used in the analysis, while for the F_3_ families, three biological replicates for each family with 20 plantlets each were analyzed.

Analysis of variance (ANOVA) of disease traits was performed to test the significance of differences between accessions and replications using the aov function in R. The effects of replications and genotypes were accounted for in the model. The values of variance obtained from the ANOVA were used to calculate broad-sense heritability according to Nyquist (1991). Correlations between traits were estimated using the Spearman coefficient.

### Genotyping of the GWAS Panel and the F_2_ Population

The accessions included in the rice panel were Genotyped-by-Sequencing (GBS) following a pipeline described by Biscarini et al. (2016), except for the number of tags required for the alignment to the Nipponbare reference sequence (1 instead of 5). A set of 246,084 SNPs were identified, mapped on the Os-Nipponbare-Reference-IRGSP-1.0 pseudomolecule assembly (Kawahara et al. 2013). The original SNP dataset was filtered with the program PLINK1 (Purcell et al. 2007) to avoid the biased detections due to rare alleles and missing data. Markers with a call rate value lower than 95% and with minimum allele frequency (MAF) lower than 5% were discarded. After filtering for call rate and MAF, a total number of 37,423 SNPs was subsequently used for the GWAS analysis. The population structure was preliminarily defined as in Volante et al. (2017b), with the same parameters. Briefly, results were crossed from Principal Component Analysis conducted using Tassel v5.2.0 (Bradbury et al. 2007), Jukes-Cantor phylogenetic clustering with MEGA7 (Kumar et al. 2016) and a Bayesian model-based analysis performed with Structure, v2.3.4 (Pritchard et al. 2000) on a subset of 9996 markers (i.e., a random selection of 833 SNPs per chromosome).

Genomic DNAs of the F_2_/F_3_ population was digested using the restriction enzyme ApeKI and bar-coded following the protocol as described by Elshire et al. (2011). Sets of 96 samples per lane were sequenced by the Institute for Genomic Diversity (Cornell University, Ithaca, NY, USA) and SNP calling was based on the Os-Nipponbare-Reference-IRGSP-1.0 pseudomolecule assembly and intersected with the genome annotation (Kawahara et al. 2013), as described above for the GWAS panel. The GBS analysis generated a total of 131,092 SNP markers. After the removal of monomorphic SNP markers across the F_2_ lines, a set of 11,745 polymorphic SNPs (8.96%) was obtained. Moreover, all the SNPs having a call rate lower than 90% and/or the ones with MAF lower than 3% were removed from the dataset. A total of 3724 high-quality SNPs were obtained.

A total of 110 mapped SSR markers (McCouch et al. 2002) listed in the Gramene markers database (http://www.gramene.org/marker) were also tested for polymorphisms between Salvo and Maratelli. The polymorphic SSRs were analyzed in the segregant population using the M13-tailed primer method (Zhou et al. 2002) to label amplicons. PCRs were carried out in a 10 μL reaction volume as described in Urso et al. (2016). Amplicons were separated using an ABI 3130xl Genetic Analyzer sequencer (Applied Biosystems), GeneScan ROX 500 was used as size standard. Visualizations and sizing of the PCR fragments were performed using the GeneMapper software version 4.0 (AppliedBiosystem). The analysis identified 46 (37.27%) polymorphic SSRs and their segregation was analyzed in the 130 F_2_ plants.

### Linkage Disequilibrium and GWAS Analysis

The computation of pairwise LD (r^2^) was performed on the marker subset used for the analysis of population structure, following the same pipeline described in Volante et al. (2017b).

A total of 37,423 SNPs were used for the GWAS analysis. A Mixed Linear Model (MLM) was applied, with the kinship matrix (K) as a random effect to consider the population stratification. Association analyses were performed for the two blast evaluations (field and growth chamber), using the average between SES scores recorded in 2013 (average of two replicates) and 2014 for field data, while the average score of the three replicates was used for the growth chamber data. The Tassel v5.2.0 software was used with the following parameters: optimal compression, genetic and residual variance estimated for each marker (P3D OFF). A *p*-value of the association to the phenotypic traits was calculated for each marker; the significance threshold to declare a marker as associated was set to 0.05 after correction for multiple testing using the false discovery rate (FDR) method according to Benjamini and Hochberg (1995). Manhattan plots and Q–Q plots of each trait were drawn using the R package qqman (Turner, unpublished). Single-SNP associations were considered as true positive only when a peak of multiple SNPs was observed, even at –log10(*p*) values below the FDR threshold, in the Manhattan plot. Clusters of SNPs in full LD showing the same *p*-value in the association analysis were considered as a peak region.

The chromosome-wise local LD was calculated by the Haploview v4.2 software (Barrett et al. 2005) as previously described (Volante et al. 2017a, 2017b). The regions defined by the peak marker/region positions including 100 Kbp upstream and downstream (corresponding to the half-maximum average LD decay estimated on the LOESS curve fitting the data) were screened to search for candidate genes underlying each trait. When a single marker was associated, this was assumed as peak. In the case of the overlapping regions between field and growth chamber experiments and showing different peak positions, the whole interval between the two was considered as a unique peak.

All gene loci within these intervals were extracted from the annotation of *Oryza sativa* reference sequence (Os-Nipponbare-Reference-IRGSP-1.03; http://rapdb.dna.affrc.go.jp/download/irgsp1.html) and reported in Additional file 6: Table S3. In order to validate the above results, all annotated gene loci included in the selected genomic regions were compared to genes known to be related to the phenotypic traits analyzed and available in the Oryzabase database (https://shigen.nig.ac.jp/rice/oryzabase/)(Additional file 6: Table S3) or in literature.

### Linkage and QTL Analyses of the F_2_ Population

The genetic linkage map was constructed with JoinMap v. 4 (van Ooijen 2006) and the Kosambi mapping function was used to calculate map distance (Kosambi 1944). Prior to mapping, from the polymorphic marker set we further removed markers showing more than 10% missing values and markers with a minor allele frequency (MAF) significantly deviating from the expected 1:1 ratio (MAF < 0.3).

The initial linkage grouping of markers was done at a LOD threshold of 5.0 and the final mapping was done by combining linkage groups that belong to the same chromosome if the distance was less than 40 cM.

The QTL discovery was performed using the R/qtl module of the R statistical package (Broman et al. 2003). A genome-wide significance level of 5% was calculated after 1000 permutations (Churchill and Doerge 1994). The position and the effect of the QTL were estimated using the multiple imputation method (Sen and Churchill 2001) by executing the “sim.geno” command, followed by the “fitqtll” and “refineqtl” commands. To search for additional QTLs, the “addqtl” command was used. When multiple QTLs were detected, “fitqtl” and “refineqtl” were reapplied to test a model containing different QTLs and their interaction effect. The confidence interval (CI) of each QTL was determined as proposed by Darvasi and Soller (1997).

## Supplementary information

**Additional file 1 Figure S1**. Structure output crossed with results from a Principal Component Analysis (PCA) and neighbor-joining tree.

**Additional file 2 Table S1**. List of accessions used in the study with their geographical origin, taxonomical group, commercial class, group assigned by the STRUCTURE analysis and blast score values.

**Additional file 3 Table S2**. Statistical analyses of the phenotypic traits: ANOVA and h^2^ for Field and Growth chamber evaluations.

**Additional file 4 Figure S2.** Analysis of the genome-wide LD decay in the rice panel used for GWAS.

**Additional file 5 Figure S3.** Plots of SNPs significantly associated (FDR > 0.05) to blast resistance loci against their chromosome positions and –log10(p) values. BRF and BRGC refer to Blast resistance in Field and Blast Resistance in Growth Chamber, respectively.

**Additional file 6 Table S3.** Gene models contained in the regions defined by the GWAS analysis, from the Os-Nipponbare-Reference-IRGSP-1.0.

**Additional file 7 Table S4**. Colocalization between MTA and known Pi genes. Bold names indicate cloned genes.

**Additional file 8 Table S5**. A. Salvo x Maratelli linkage map. Linkage Groups (LG), chromosomes, marker names and their position (cM, obtained using the Kosambi mapping function) are reported. B. Distribution of molecular markers in the chromosomes of the Salvo x Maratelli map. C. QTL detected in Salvo x Maratelli segregating population. LOD score, percentage of phenotypic variance explained (R2), additive effect and the confidence interval (CI; Darvasi and Soller,^1997^) are reported.

## Data Availability

All data generated or analyzed during this study are included in this published article [and its supplementary information files].

## Abbreviations

ABC: ATP-Binding Cassette
ANOVA: Analysis Of Variance
BRF: Blast Resistance in Field
BRGC: Blast Resistance in Growth Chamber
cv: Cultivar
FDR: False Discovery Rate
GWAS: Genome-Wide Association Study
LD: Linkage Disequilibrium
LOD: Logarithm Of Odds
LOESS: Locally Estimated Scatterplot Smoothing
MAF: Minor Allele Frequency
MATH: Meprin And TRAF Homology
MLM: Mixed Linear Model
MTA: Marker-Trait Association
NB-ARC: Nucleotide-Binding APAF-1 R proteinCED-4
NB-LRR: Nucleotide Binding-Leucine Rich Repeat
P3D: Population Parameters Previously Determined
PCA: Principal Component Analysis
PCR: Polymerase Chain Reaction
Q–Q plot: Quantile-Quantile Plot
QTL: Quantitative Trait Locus
RING: Really Interesting New Gene
SES: Standard Evaluation System
SNP: Single Nucleotide Polymorphism
SSR: Simple Sequence Repeat
SxMBRQTL: Salvo x Maratelli Blast Resistance Quantitative Trait Locus
