# Supplementary material for: Genome wide association studies for japonica rice resistance to blast in field and controlled conditions
Source: Rice (N Y). 2020 Oct 8;13:71. doi: 10.1186/s12284-020-00431-2 (PMC7544789; doi:10.1186/s12284-020-00431-2)
Supplement: Supplementary file 1 — Additional file 1 Figure S1. Structure output crossed with results from a Principal Component Analysis (PCA) and neighbor-joining tree. [file 12284_2020_431_MOESM1_ESM.pdf]

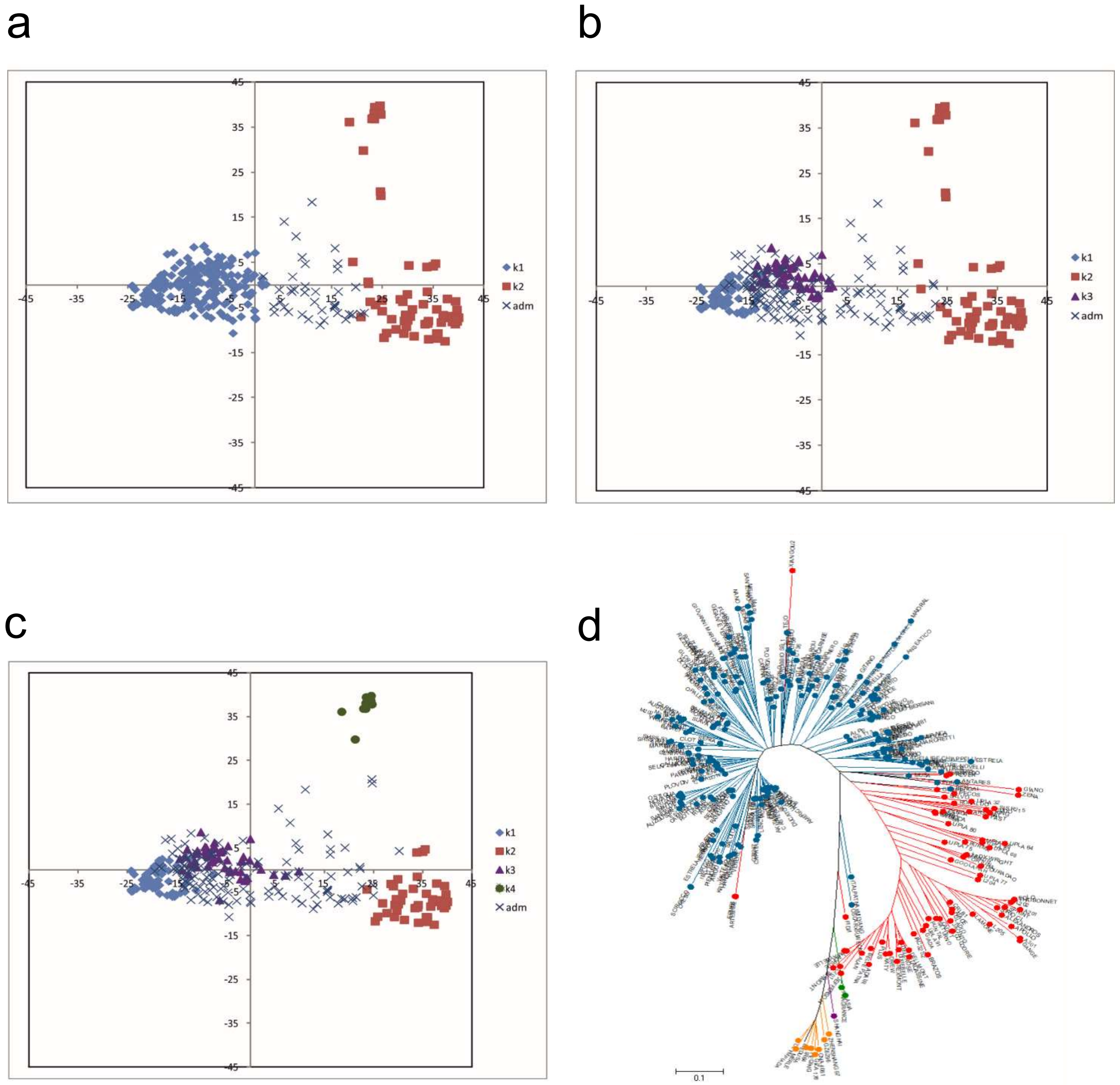


**Additional file 1: Figure S1.** Structure output crossed with results from a Principal Component Analysis (PCA) and neighbor-joining tree: a) K=2 separate temperate *japonica* (K1) from tropical *japonica* (K2), with 39/311 of admixed (12.5%); b) K=3 discriminate, within temperate *japonica*, longA type (K3) and the round medium type (K1), with 129/311 of admixed (41,5%); c) K=4 separate the *indica* (K4) from tropical *japonica*, with 136/311 of admixed (43,7%); d) Jukes and Cantor model-based neighbor-joining tree of the panel. yellow = *indica*; green = aromatic; violet = aus; red = tropical *japonica*; blue = temperate *japonica*
