# Supplementary material for: Genome wide association studies for japonica rice resistance to blast in field and controlled conditions
Source: Rice (N Y). 2020 Oct 8;13:71. doi: 10.1186/s12284-020-00431-2 (PMC7544789; doi:10.1186/s12284-020-00431-2)
Supplement: Supplementary file 4 — Additional file 4 Figure S2. Analysis of the genome-wide LD decay in the rice panel used for GWAS. [file 12284_2020_431_MOESM4_ESM.pdf]

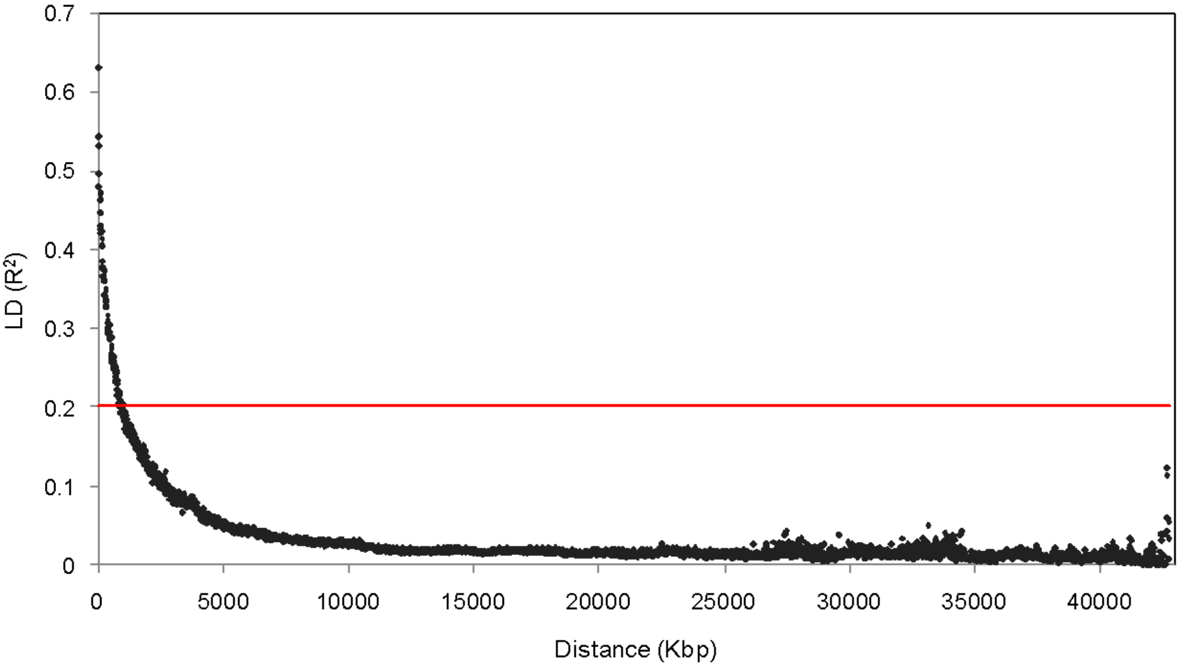

Chromosome	#SNP	Chr. size (bp)*		SNP density (bp/SNP)	Critical decay distance (bp)
1	4,346	43,270,923		9,956	665,000
2	3,044	35,937,250		11,806	675,000
3	2,429	36,413,819		14,991	955,000
4	3,145	35,502,694		11,289	1,285,000
5	2,271	29,958,434		13,192	1,205,000
6	2,778	31,248,787		11,249	525,000
7	2,935	29,697,621		10,118	1,145,000
8	3,259	28,443,022		8,728	1,125,000
9	1,787	23,012,720		12,878	815,000
10	3,904	23,207,287		5,944	1,355,000
11	4,641	29,021,106		6,253	445,000
12	2,884	27,531,856		9,546	1,015,000
Total	37,423	373,245,519	Average	10,496	934,167

* according to Kawahara et al., 2013

Additional file 4: Figure S2. Analysis of the genome-wide LD decay in the rice panel used for GWAS. The curve represents LD averaged values in 10 kb windows (see Materials and Methods for details). The red line represents the threshold R_2 value to consider two markers as unlinked. The table reports, for each chromosome, the number of SNPs, the size, the marker density and distances corresponding to a R_2 value of 0.2. The average genome-wide value is also shown.
