# Supplementary material for: Genome wide association studies for japonica rice resistance to blast in field and controlled conditions
Source: Rice (N Y). 2020 Oct 8;13:71. doi: 10.1186/s12284-020-00431-2 (PMC7544789; doi:10.1186/s12284-020-00431-2)
Supplement: Supplementary file 5 — Additional file 5 Figure S3. Plots of SNPs significantly associated (FDR > 0.05) to blast resistance loci against their chromosome positions and –log10(p) values. BRF and BRGC refer to Blast resistance in Field and Blast Resistance in Growth Chamber, respectively. [file 12284_2020_431_MOESM5_ESM.pdf]

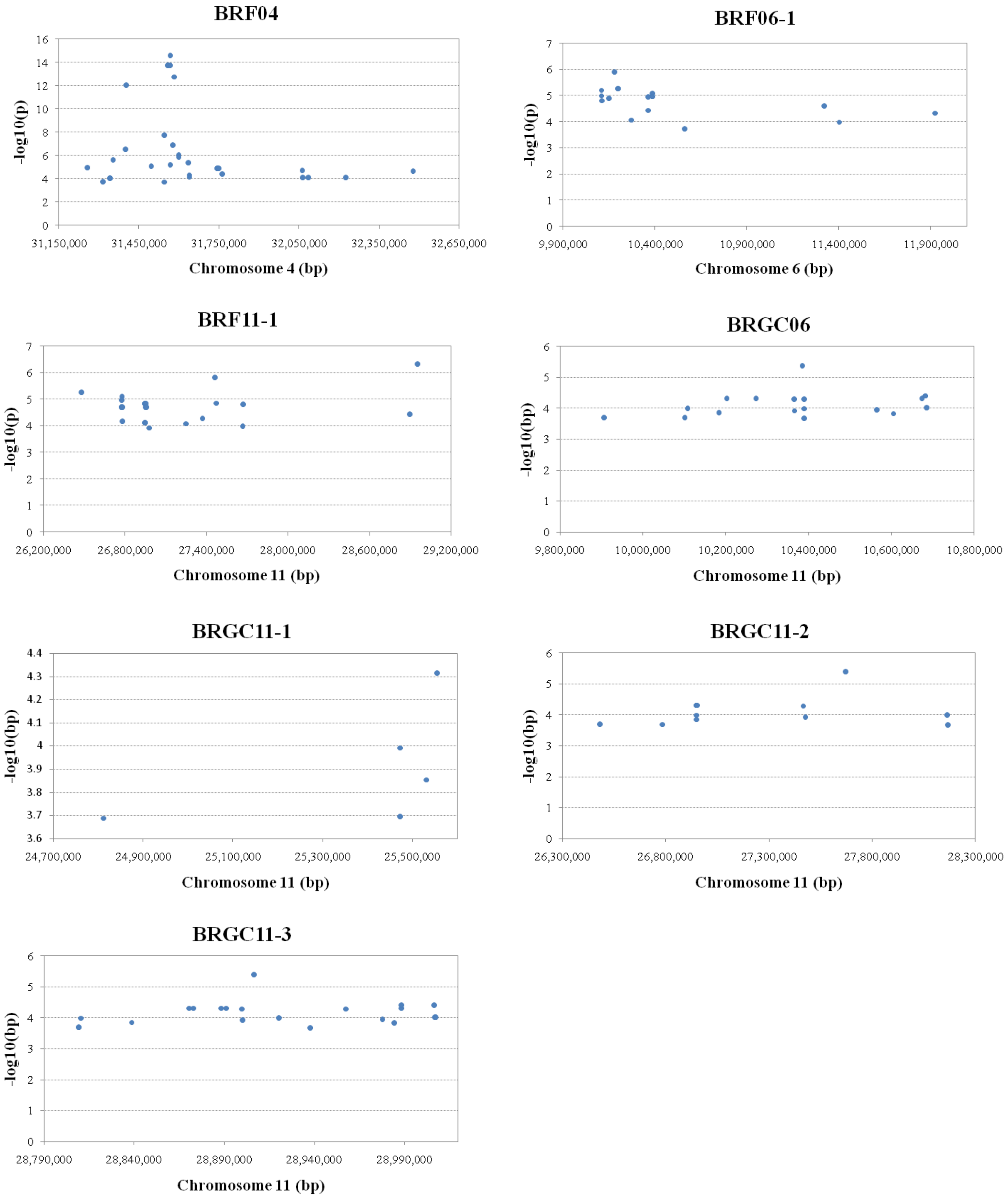


**Additional file 5: Figure S3.** Plots of SNPs significantly associated (FDR>0.05) to blast resistance loci against their chromosome positions and –log10(p) values. BRF and BRGC refer to Blast resistance in Field and Blast Resistance in Growth Chamber, respectively.
